# Prevalence and outcomes of rapidly progressive dementia: a retrospective cohort study in a neurologic unit in China

**DOI:** 10.1186/s12877-023-03841-1

**Published:** 2023-03-14

**Authors:** Xiaoyan Liu, Yan Sun, Xuyan Zhang, Ping Liu, Kan Zhang, Lihua Yu, Yujie Su, Yuan Yuan, Qing Ke, Guoping Peng

**Affiliations:** 1 Department of Neurology, the First Affiliated Hospital, Zhejiang University School of Medicine, Qingchun Road No.79, 310009 Hangzhou, China; 2 Department of Neurology, Haining People’s hospital, Jiaxing, China

**Keywords:** Alzheimer’s disease, Carbon monoxide poisoning, Dementia with Lewy bodies, Neurosyphilis, Rapidly progressive dementia

## Abstract

**Background:**

Rapidly progressive dementia (RPD) is a syndrome originating from various diseases. Recent advances have allowed a better understanding of its categories and spectrum; however, it remains challenging to make an accurate differential diagnosis and prognosis prediction.

**Methods:**

This study was a retrospective evaluation of all participants admitted to the neurology department of a single center in China from January 2015 to December 2019. The screened patients met the RPD criteria and their characteristics were collected to explore a diagnostic pattern of RPD. In addition, outcomes of RPD were evaluated with the Glasgow Outcome Scale (GOS), activities of daily living scale (ADL), and simplified Mini-Mental State Examination (MMSE), and different prognostic analysis methods were performed to determine the prognostic factors of RPD.

**Results:**

A total of 149 RPD patients among 15,731 inpatients were identified with an average MMSE value of 13.0 ± 4.6 at baseline. Etiological epidemiology revealed infectious, neurodegenerative and toxic/metabolic diseases as the three largest groups, accounting for 26.2%, 20.8% and 16.8% of all cases, respectively. In particular, prevalence rates of Creutzfeldt–Jakob disease (13.4%), Alzheimer’s disease (11.4%), carbon monoxide poisoning (8.1%), neurosyphilis (5.4%) and dementia with Lewy bodies (5.4%) were highest in this series. A recommended diagnostic framework for RPD etiology was thus established. Follow-up evaluations showed a negative correlation between age and GOS scores (r=-0.421, P < 0.001), as well as age and simplified MMSE scores (r_s_ =- 0.393, P < 0.001), and a positive correlation between age and ADL scores (r_s_ =0.503, P < 0.001), and significantly different GOS, ADL and simplified MMSE scores across various etiologies (P = 0.003; F = 9.463, *P* < 0.001; F = 6.117, *P* < 0.001).

**Conclusion:**

Infectious, neurodegenerative and toxic-metabolic entities were the most common RPD categories, and establishing a practical approach to RPD etiology would allow better disease management.

## Introduction

Rapidly progressive dementia (RPD) is a syndrome that usually develops over months, weeks or days. Diverse categories have been identified, including those related to neurodegenerative, vascular, toxic-metabolic, infectious, autoimmune, paraneoplastic, and neoplastic entities [1, 2]. In case that many types of RPD are treatable and other conditions result in poor outcomes or may be fatal, it is paramount to better understand the categories and spectrum of RPD to provide a timely and accurate diagnosis [3]. To our knowledge, several studies have focused on RPD categories and characteristics, especially with Creutzfeldt–Jakob disease (CJD) [4], and improved magnetic resonance imaging (MRI) techniques and discoveries of many new autoimmune antibodies and infectious causative agents have avoided some diagnostic pitfalls [5].

To be noted, much effort has been made to explore the epidemiology and characteristics of RPD widely in recent years. A retrospective clinico-pathological study from Spain revealed that prion diseases represented the most frequently observed group (67%) [6], while a study from a tertiary care medical center in Spain and suggested CJD frequency was related to the referral differences across specialized centers [7]. Another study included 104 RPD patients in Argentina, 27.9% of chronic neurodegenerative RPD and 72.1% of non-chronic neurodegenerative RPD, and found that assessment on time to dementia, brain MR, and cerebrospinal fluid (CSF) could identify those diseases [8]. Besides, a study performed an evaluation on 47 RPD patients referred to Attikon University Hospital, Greece, and revealed that neurodegenerative diseases were the most frequent diagnosis (38%), followed by prion disease (19%) and autoimmune encephalopathy (17%) [9]. However, another study performed in a Greek Tertiary Referral Center in Athens on 68 RPD patients suggested that secondary dementia is the most frequent causes [10]. Similarly, a retrospective case analysis on 187 RPD patients in India revealed a most common cause of infections (39%) [11], and data from a neurologic unit of a tertiary hospital in Brazil also took immune-mediated disease as the most common etiology (45.9%) [12]. Furthermore, an outpatient memory clinic assessed 96 suspected RPD patients, and found atypical presentations of common neurodegenerative dementia was in a proportion of 90% among 96 suspected RPD patients [13]. However, it remains challenging to make a rapid and accurate differential diagnosis. In addition, valid data on RPD-related factors and outcomes were only partially available.

Here, we conducted a retrospective cohort study with RPD patients from a single center in southern China over 5 years to enhance knowledge of the epidemiology and create a classification scheme in China. Moreover, we performed a longitudinal follow-up of these participants, determined the disease courses and explored risk factors.

## Materials and methods

### Subjects

**Fig. 1:**
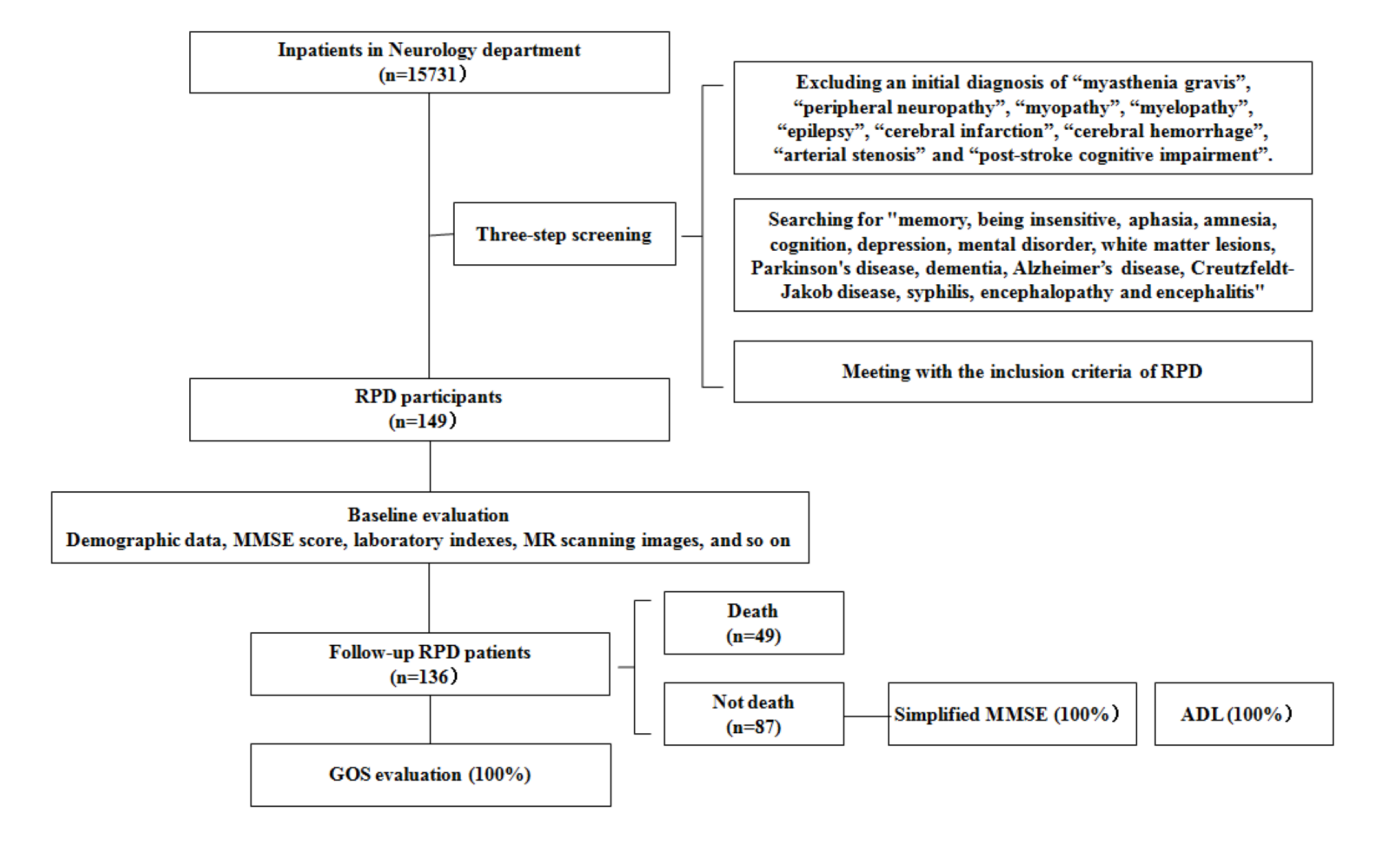
Enrollment, evaluation and follow-up of patients with RPD in a single center

This study involved a retrospective evaluation of RPD patients by referring to a database including all participants hospitalized in the Neurology Department of the First Affiliated Hospital, Zhejiang University School of Medicine, in Southern China between January 2015 and December 2019 and identifying RPD patients. Inclusion criteria were as follows: (1) chief complaints of cognitive decline and progression to dementia with Mini-Mental State Examination (MMSE) scores < 20 or inability to respond within 2 years; or (2) rapidly progressive Alzheimer’s disease (rpAD) with a loss of ≥ 6 points per year [14]. In particular, participants diagnosed with poststroke cognitive impairment were excluded. To improve manageability, this screening task was divided into three steps. Demographic data, neuropsychological data, laboratory indexes and MRI data were collected at baseline. Moreover, a follow-up evaluation was designed to be performed 1 year after discharge from the hospital. A simple flow chart of this study is shown in Fig. 1.

### Diagnosis

Medical records were checked for each included patient, including demographic data, neuropsychological data such as MMSE scores, laboratory indexes and MRI data. Laboratory indexes included routine blood examination, serum electrolytes, blood glucose, liver and renal function, acute C-reactive protein (CRP), erythrocyte sedimentation rate (ESR), antinuclear antibody, coagulation function, D-dimer level, syphilis and HIV antibodies, homocysteine, HbA1c, folic acid, vitamin B12, and thyroid function. In addition, the included patients were all assessed by brain MRI [T1, T2, fluid-attenuated inversion recovery (FLAIR)/diffusion-weighted imaging (DWI)] and selectively evaluated by electroencephalography (EEG), routine CSF testing, CSF 14-3-3 testing, serum or CSF causative agents, and immunity indicators. All these data were obtained from the database.

Probable AD was defined according to the National Institute on Aging-Alzheimer’s Association criteria [15]. Dementia with Lewy bodies (DLB) was defined according to the consensus report of the DLB consortium [16]. Frontotemporal lobar degeneration (FTLD) was diagnosed using consensus criteria accompanied by imaging evidence [17]. Vascular cognitive impairment was defined according to the relevant criteria [18]. This study adopted the clinical diagnostic criteria of sporadic CJD [19]. Autoimmune encephalopathy/encephalitis was determined by the detection of specific serum and/or CSF antibodies.

### Clinical evaluation

The MMSE scale was used to determine general cognitive function at baseline. For a quantitative evaluation of participant outcomes in the follow-up, the Glasgow Outcome Scale (GOS) score was calculated to determine the outcome as follows: 1, death; 2, vegetative state; 3, severe disability and demanding care in life; 4, mild disability and ability to work under protection; and 5, return to normal life. Specifically, two methods were adopted: one was GOS binary classification: good outcome (score = 4 or 5) versus poor outcome (score = 1, 2 or 3), and the other was to divide patient outcomes into five subgroups according to GOS score 1 to 5. The 14-item version of the activity of daily living scale (ADL) and simplified version of the MMSE were assessed among patients with GOS scores above 1.

### Statistical analysis

Statistical analyses were performed by SPSS (version 24.0). Categorical variables are presented as numbers (proportions), and continuous variables are presented as the median (quartile1-quartile3) or mean ± standard deviation, including age, length of hospital stay, baseline MMSE score, and various laboratory indexes in these participants. The differences in baseline MMSE scores, age and accessory data among groups with distinct etiologies were compared by analysis of variance, and education differences were tested with the chi-square test. For the prognostic analysis, Spearman’s test was initially used to calculate the correlation between continuous independent variables such as age, laboratory indexes, or baseline MMSE score, with the outcome index of GOS 1 to 5, ADL score or simplified MMSE score. For the categorical etiological variable, whereas a linear trend test was used to calculate its correlation with GOS scores, analysis of variance was used for its correlation with ADL or simplified MMSE scores. In addition, multivariate logistic regression model analyses were further performed, while age, sex, and baseline MMSE score were included as the independent variables, and the GOS binary classification level was taken as the outcome index. A P value < 0.05 was considered statistically significant.

## Results

### Demographic, clinical and accessory data of RPD participants at baseline

**Fig. 2:**
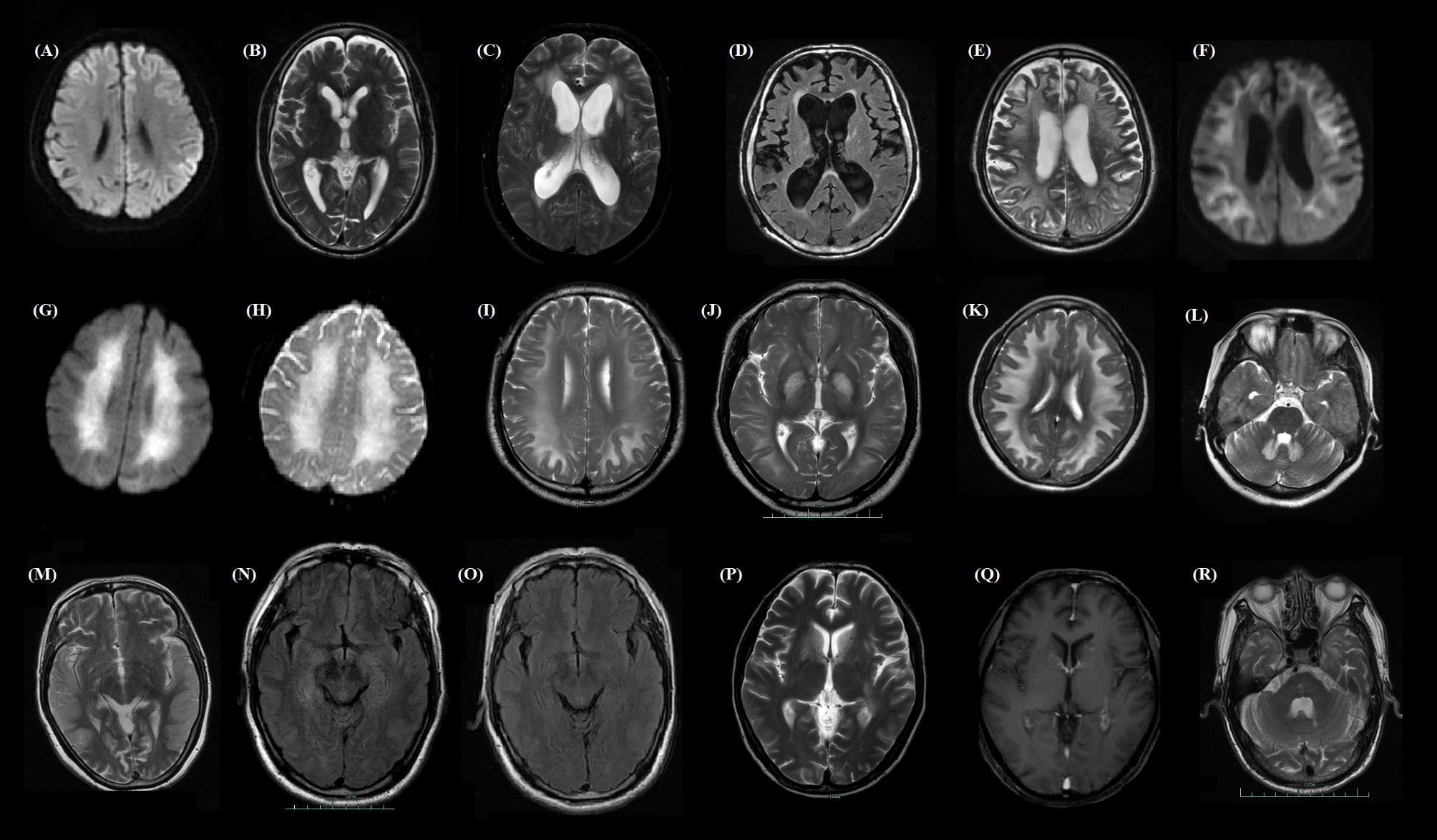
Selected brain MRI images in participants with rapidly progressive dementia. The descriptive text of each image is shown with definitions of disease name, age, sex, disease course, and MRI data. **(A) Creutzfeldt–Jakob disease**: 72 years, female, 20 days. DWI image indicating the bilateral frontal and parietal cortex hyperintensities. **(B) Neurosyphilis**: 61 years, male, 2 months. T2WI demonstrating wide brain atrophy. **(C) Tuberculous meningoencephalitis**: 55 years, male, 1 month, T2WI demonstrating ventricular enlargement, paraventricular hyperintensities and increased vascular shadow. **(D) Alzheimer’s disease**: 75 years, male, 1 year, T2WI demonstrating wide brain atrophy and ventricular enlargement. **(E & F) Neuronal intranuclear inclusion disease**: 78 years, male, 2 months, T2WI demonstrating diffuse white matter hyperintensities and brain atrophy, and DWI images indicating hyperintensities involving corticomedullary junction in the frontal and parietal lobes. **(G & H): CO poisoning**: 61 years, female, 1 month, DWI and ADC images indicating diffuse sub-cortical hyperintensities. **(I & J) Polyvinyl alcohol poisoning**: 35 years, male, 1 month, T2WI images showing diffuse subcortical and globus pallidus hyperintensities. **(K & L) Dichloroethane poisoning**: 34 years, female, 10 days, T2WI showing diffuse subcortical and dentate nucleus hyperintensities. **(M) Mitochondrial encephalomyopathy with lactic acidosis and stroke-like episode**: 52 years, female, half a month. T2WI showing hyperintensity and local swelling on the bilateral temporal lobes. **(N & O) Autoimmune encephalitis associated with LGI1 receptor**: 47 years, male, 20 days, FLAIR showing hyperintensities on the bilateral hippocampus (N), and lesions disappeared after treatment at 6-month follow-up (O). **(P & Q) Sjogren’s syndrome with central nervous system involvement**: 45 years, male, 2 months, T2WI showing bilateral basal ganglia (caudate nucleus, globus pallidus and anterior thalamus) symmetrical hyperintensities (P), and enhanced sequence showing significantly enhanced lesions in patches (Q). **(R) Cerebral autosomal dominant arteriopathy with subcortical infarcts and leukoencephalopathy**: 62 years, male, 2 years, T2WI images showing hyperintensity on the pole of the bilateral temporal lobes

A conclusive diagnosis was made in a total of 149 participants with RPD among 15,731 inpatients, with proportions of 16.8%, 18.8%, 9.396%, 20.1%, and 34.9% from 2015 to 2019, respectively. There were 81 males and 68 females, with an average age of 58.6 ± 15.0 years, an average length of hospital stay of 13.7 ± 8.8 days, and an average MMSE score at baseline of 13.0 ± 4.6. In addition, education levels were diverse, including 55.7% with more than 6 years of education, 25.5% with 1 to 6 years of education, 15.4% with no education, and 3.4% did not have this information available. Furthermore, MRI images were investigated as a core evaluation, which was critical to reach a quick initial diagnosis and differential diagnosis, and selected brain MRI images in these participants are shown in Fig. 2.

### Etiological epidemiology among RPD participants

The etiological distribution of RPD participants was determined in this cohort: 31 patients had a cause related to neurodegenerative entities; 39, infectious; 13, vascular; 5, mixed; 15, inflammatory; 25, toxic/metabolic; 5, neoplastic; 5, unknown; and 11, other entities. That is, the three largest groups were infectious, neurodegenerative diseases and toxic/metabolic diseases, accounting for 26.2%, 20.8% and 16.8% of all cases, respectively. These etiologies were consistently present in a high proportion from 2015 to 2019, followed by inflammatory and vascular entities. Moreover, the etiological distribution was further calculated by grouping together the overall data over 5 years, which revealed that AD was the most common neurodegenerative disease, followed by DLB, neuronal intranuclear inclusion disease and FTLD, while CJD was the most common infectious disease, followed by neurosyphilis. It is worth noting that a high percentage of toxic/metabolic entities was demonstrated in this cohort, especially carbon monoxide (CO) poisoning. For the inflammatory category, this study contained 7 patients with autoimmune encephalitis associated with AMPA2, LGI1 or GABAB receptors. A detailed etiological classification of RPD participants in this study is shown in Table 1.

**Table 1:** Etiological classification of rapidly progressive dementia participants in this study

Etiology	Number (%)	Age (years)	Education level	Baseline MMSE value
**Infectious entities**	**39 (26.2%)**	**56.1 ± 14.5**	**6, 9, 22**	**13.1 ± 4.6**
Creutzfeldt-Jakob disease	20 (13.4%)			
Neurosyphilis	8 (5.4%)			
Other infections entities	11 (7.4%)			
**Neurodegenerative entities**	**31 (20.8%)**	**68.4 ± 10.8**	**8, 6, 16**	**11.2 ± 4.0**
Alzheimer’s disease	17 (11.4%)			
Dementia with Lewy body	8 (5.4%)			
Neuronal intranuclear inclusion disease	4 (2.7%)			
Frontotemporal lobar degeneration	2 (1.4%)			
**Toxic/metabolic entities**	**25 (16.8%)**	**53.1 ± 12.7**	**3, 3, 18**	**12.4 ± 5.5**
Carbon monoxide poisoning	12 (8.1%)			
Heroin poisoning	1 (0.7%)			
Dichloroethane poisoning	1 (0.7%)			
Alcohol poisoning	2 (1.3%)			
Other toxic entities	2 (1.3%)			
Mitochondrial encephalopathy	2 (1.3%)			
Wernicke encephalopathy	1 (0.7%)			
Hashimoto encephalopathy	1 (0.7%)			
Radiation encephalopathy	1 (0.7%)			
Other metabolic entities	2 (1.3%)			
**Inflammatory entities**	**15 (10.1%)**	**51.1 ± 13.7**	**1, 5, 8**	**13.8 ± 4.6**
Anti-AMPA2 receptor encephalitis	3 (2.0%)			
Anti-LG1 receptor encephalitis	3 (2.0%)			
Limbic encephalitis	2 (1.3%)			
Anti-GABAB receptor encephalitis	1 (0.7%)			
Central demyelinating disease	2 (1.3%)			
Sjogren’s syndrome with central involvement	1 (0.7%)			
Other inflammatory entities	3 (2.0%)			
**Vascular entities**	**13 (8.7%)**	**63.1 ± 12.8**	**1, 5, 7**	**14.6 ± 4.3**
**Other entities**	**11 (7.4%)**	**55.0 ± 17.4**	**2, 3, 6**	**15.0 ± 3.6**
**Mixed entities**	**5 (3.4%)**	**72.6 ± 9.1**	**1, 3, 1**	**12.2 ± 6.5**
**Neoplastic entities**	**5 (3.4%)**	**53.8 ± 13.1**	**0, 3, 2**	**14.3 ± 2.1**
**Undetermined entities**	**5 (3.4%)**	**54.8 ± 27.1**	**1, 1, 3**	**16.0 ± 1.0**

Note: Age and baseline MMSE value are presented as mean ± standard deviation, education level is presented as numbers of no education, 1 to 6 years of education and more than 6 years of education

Differences in the distributions of age and education level across etiologies were calculated and it revealed a significant difference in age (F = 4.123, *P* < 0.001) but no difference in education level. Similarly, no significant differences were observed in baseline MMSE scores. This study also performed a difference analysis of laboratory indexes across etiologies, including hemoglobin, uric acid, CRP, ESR, D-dimer, homocysteine, HbA1c, folic acid and vitamin B12, and merely showed significant differences in vitamin B12 (F = 2.603, *P* = 0.012) and CRP (F = 2.176, *P* = 0.035) but not the other 7 indexes.

### Prognostic factors in RPD participants

#### Analyses of prognostic indicators calculated by GOS

While continuous variables such as age, hemoglobin, uric acid, CRP, ESR, D-dimer, homocysteine, HbA1c, folic acid, vitamin B12, and baseline MMSE score were individually considered independent variables, Spearman testing was used to analyze their correlation with the GOS multicategory score. As a result, there was a negative correlation between age and GOS score (r=-0.421, *P* < 0.001), but there were no statistically significant correlations between other factors and GOS scores.

On the other hand, a linear trend test was used to calculate the correlation between categorical variables of etiological factors and GOS, and a significance level of *P* = 0.003 was demonstrated, indicating that diverse etiologies were associated with diverse outcomes. Specifically, the average proportion of patients who died or developed a vegetative state was 36.8% in all included participants, which included (in descending order of proportion) the following etiologies: neoplastic, mixed, infectious, unknown, vascular, degenerative, inflammatory, toxic/metabolic and other entities. Moreover, the incidence of mild disability or return to normal life was in descending order including inflammatory, other, toxic/metabolic, infectious, unknown, vascular, degenerative, neoplastic and mixed entities. Namely, those in the inflammatory and toxic/metabolic categories obtained relatively good outcomes. The details of the GOS distribution among RPD patients across each etiology are shown in Table 2.

**Table 2:** GOS distribution among RPD patients for each etiology

Etiology	GOS distribution, Number (%)				
	GOS = 1/2 Death/vegetative state	GOS = 3 Severe disability, demanding care in life	GOS = 4 Mild disability, able to work under a protection	GOS = 5 Return to normal life	Total
Neurodegenerative	8 (29.6%)	17 (63.0%)	2 (7.4%)	0 (0.0%)	27
Infectious	21 (56.8%)	3 (8.1%)	6 (16.2%)	7 (18.9%)	37
Vascular	4 (30.8%)	7 (53.8%)	2 (15.4%)	0 (0.0%)	13
Mixed	3 (60.0%)	2 (40.0%)	0 (0.0%)	0 (0.0%)	5
Inflammatory	4 (28.6%)	1 (7.1%)	2 (14.3%)	7 (50.0%)	14
Toxic/metabolic	2 (10.5%)	7 (36.8%)	0 (0.0%)	10 (52.6%)	19
Neoplastic	5 (100.0%)	0 (0.0%)	0 (0.0%)	0 (0.0%)	5
Undetermined	2 (40.0%)	2 (40.0%)	1 (20.0%)	0 (0.0%)	5
other	1 (9.1%)	3 (27.3%)	1 (9.1%)	6 (54.5%)	11
Total	50 (36.8%)	42 (30.9%)	14 (10.3%)	30 (22.1%)	136

Abbreviations: Glasgow outcome scale (GOS), Rapidly progressive dementia (RPD)

#### Analysis of prognostic indicators calculated by ADL and simplified MMSE scores

To better explore prognostic indicators, this study used ADL and simplified MMSE scores to evaluate outcomes in participants with a GOS score above 1, when those longitudinal ADL or simplified MMSE scores were available. While continuous variables such as age, hemoglobin, uric acid, CRP, ESR, D-dimer, homocysteine, HbA1c, folic acid, vitamin B12, and baseline MMSE measures were individually considered independent variables, Spearman testing was used to analyze their correlation with longitudinal ADL or simplified MMSE scores. The results showed a positive correlation between age and ADL scores (r_s_=0.503, *P* < 0.001), as well as a negative correlation between age and simplified MMSE scores (r_s_=-0.393, *P* < 0.001). Furthermore, a significant correlation was demonstrated between baseline MMSE scores and longitudinal simplified MMSE scores (r_s_=0.252, *P* = 0.025).

This study further described the correlation between etiological factors as a categorical variable and ADL scores, which resulted in significantly different ADL scores across various etiologies (F = 9.463, *P* < 0.001); average ADL scores across etiologies were, in descending order, mixed, degenerative, vascular, unknown, infectious, toxic/metabolic, other, and inflammatory categories. Similarly, a correlation between etiological factors and MMSE simplified version scores was explored, which showed significant differences in simplified MMSE scores across various etiologies (F = 6.117, *P* < 0.001); average simplified MMSE scores across etiologies were, in ascending order, mixed, degenerative, vascular, unknown, infection, toxic/metabolic, other, and inflammatory categories. It is worth mentioning that the follow-up data with the prognostic indicators measured by the ADL and simplified MMSE scores were consistent.

#### Multivariate logistic regression analysis of predictor variables in RPD

To investigate the effects of predictor variables on binary outcomes calculated by GOS, age, sex and baseline MMSE score were all included in the multivariate logistic regression model. This analysis indicated that age and baseline MMSE score were prognostic factors of RPD (*P* < 0.001 and 0.025); however, sex was not a prognostic factor of outcomes. After adjusting for other factors, for the incidence of severe disability, vegetative state, or death, each additional year of age would increase the incidence by 7.2%, and each 1-point increase in the baseline MMSE score would reduce the incidence by 11.1%. See the details in Table 3.

**Table 3:** Multivariate logistic regression analysis of RPD patients with GOS binary classification as outcome

Variable	B	S.E.	Wald	P value	OR	95%CI (lower, upper)
Age (years)	0.070	0.017	16.355	< 0.001	1.072	(1.037, 1.109)
Sex (female as a reference)	0.548	0.453	1.464	0.226	1.73	(0.712, 4.203)
Baseline MMSE value	-0.117	0.052	5.024	0.025	0.889	(0.803, 0.985)
Constant	-2.124	1.227	2.997	0.083	0.12	/

Abbreviations: Glasgow outcome scale (GOS), Mini-Mental State Examination (MMSE), Rapidly progressive dementia (RPD)

### Detailed description of participants with RPD originating from CO poisoning

The current study identified 12 patients with RPD originating from CO poisoning, accounting for 8.1% of all the etiologies and 48% of the toxic/metabolic category, the proportion of which was preceded only by CJD and AD. Among these patients, there were 5 males and 7 females, and the average age was 57.67 years old. Eight out of the 12 patients had a history of exposure to burning charcoal in a confined space, 2 patients were subsequently confirmed by serum carboxyhemoglobin testing, and the other 2 patients were diagnosed based on additional brain MRI images. In addition, except for unavailable brain MR images for 1 patient, typical brain images in those who experienced CO poisoning were revealed in the remaining 11 patients. The time interval from disease onset to our hospital visit was 5 days for 1 patient, approximately 1 month for 9 patients, approximately 2 months for 1 patient, and approximately 3 months for 1 patient. The main clinical manifestations included an early onset of headache, body aches, consciousness impairment, seizure, general malaise, tremor, delayed-onset cognitive decline, memory loss, comprehension impairment, unresponsiveness, apathy, decreased active speech, reduced physical activity and so on.

### Recommended diagnostic framework of RPD etiology in line with local conditions

**Fig. 3:**
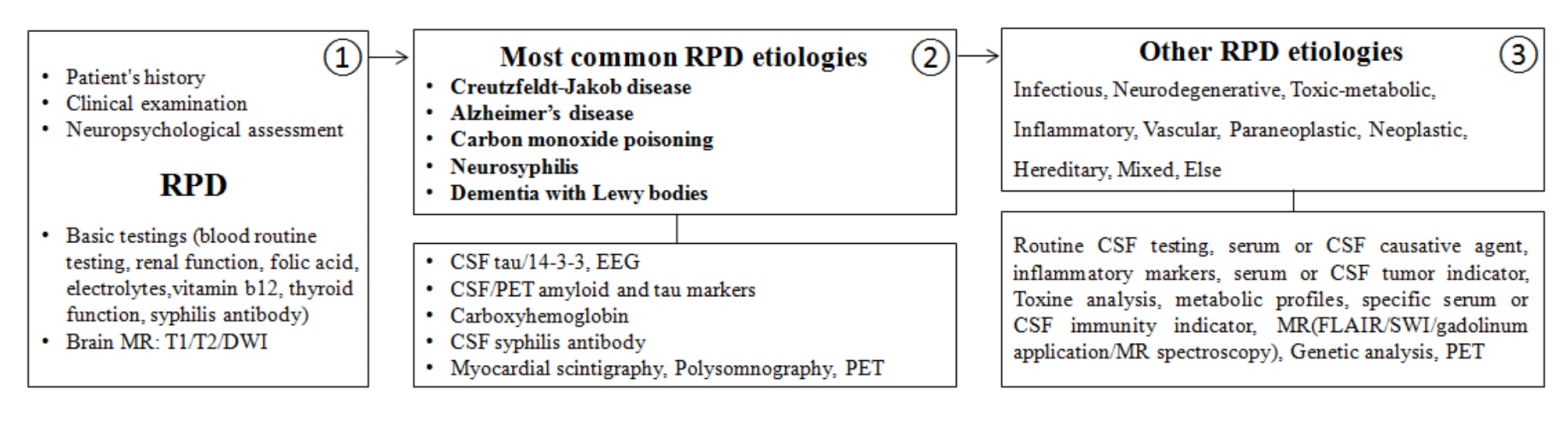
Recommended diagnostic framework of RPD etiology

Based on the epidemiological findings, this study proposes a recommended RPD diagnostic framework in line with the local conditions. First, clinical interviews, physical examinations, and overall and multidimensional cognitive testing were carried out in succession to identify RPD in these individuals, and basic testing involving routine blood testing, renal function, folic acid, electrolytes, vitamin B12, thyroid function, syphilis antibodies and MRI scanning was ordered. Second, we prioritized screening the five most common types of disease and detected specific indexes, such as CSF tau/14-3-3 protein and EEG for CJD, CSF or positron emission tomography (PET) AD markers for AD, CO hemoglobin concentration for CO poisoning, CSF treponemal antibodies for neurosyphilis, myocardial scintigraphy, polysomnography, FDG-PET or DAT-PET for DLB, and further genetic testing if necessary. If not determined, the next step is to conduct a broad exploration based on the principle of “MIDNIGHTS” (M = Metabolism, I = Inflammation/Immune, D = Degeneration, N = Neoplasm, I = Infection, G = Gland, H = Hereditary, T = Toxication/Trauma, S = Stroke). Namely, routine CSF testing, serum or CSF pathogen and inflammatory marker detection are recommended for a probable category of an infectious disease; PET and genetic testing for a neurodegenerative disease; inflammatory markers, specific toxicity analyses or metabolic markers for a toxic/metabolic disease; routine CSF testing, inflammatory markers, and specific serum or CSF autoimmune markers for an inflammatory/immune disease; and serum or CSF tumor markers, enhanced MR, MR spectroscopy, and PET for a paraneoplastic or neoplastic disease. In addition to the above diseases, an individualized plan should be designed for mixed, idiopathic, or some undetermined etiologies. See the details in Fig. 3.

## Discussion

This study enrolled RPD patients admitted to the neurology department of a comprehensive hospital in Southern China over the past five years. An epidemiological analysis demonstrated a relatively high proportion of infectious, neurodegenerative and toxic/metabolic entities, and the most common five types of disease were the following: CJD, AD, CO poisoning, neurosyphilis and DLB. Epidemiological and clinical differences among various RPD etiologies were explored. In addition, a longitudinal analysis of prognostic factors of RPD patients was performed; as a result, a relationship between age and etiological factors and the outcomes was indicated. Furthermore, one of the highlights of this study lies in establishing a practical approach to the diagnosis of RPD patients in China.

According to the current epidemiological analysis, the most common causative factor resulting in RPD was infection, and the largest group was CJD, which matched previous studies. Namely, although diverse definitions and diagnostic criteria have been used, most observations suggest that sporadic CJD is not a very rare disease and is the most common type of RPD followed by AD [1], especially among patients referred to prion disease centers, with a high prevalence ranging from 51 to 76% [7, 20]. According to a study performed in Chinese patients, the positive rates based on MRI abnormalities, CSF 14-3-3 protein and EEG periodic sharp waves were 96%, 64% and 50%, respectively [21], suggesting MR as an initial evaluation. Here, it is worth mentioning that MR images are not unique but useful, in that they do not determine but direct toward the final diagnosis. MRI was sensitive to identifying CJD with hyperintensities involving the frontal lobe followed by the parietal and temporal cortical lobes in addition to the thalamus, striatum, cerebellum and hippocampus [22], and MRI was sensitive to identifying anti-LG1 receptor encephalitis with an initial show of hyperintensities and swelling in the hippocampus or amygdala or a few lesions with a tendency toward structural atrophy at a longitudinal stage [23, 24]. Although the findings are quite broad for making a differential diagnosis based on white matter lesions, the distribution and some imaging signs could help narrow the definition [25]. In Fig. 2, white matter lesions were demonstrated in neuronal intranuclear inclusion disease, CO poisoning, polyvinyl alcohol poisoning and dichloroethane poisoning, but the clue to the diagnosis mainly relied on a combination of the DWI sequence, lesion location other than the lateral ventricle, and a tendency toward having a progressing lesion. For instance, CO poisoning corresponded to symmetrical subcortical white matter, globus pallidus or other nucleus involvement, or symmetrical frontal parietal lobe involvement, showing hyperintensity on DWI and hypointensity transforming to hyperintensity on apparent diffusion coefficient (ADC) images during the disease stage [26].

Degenerative diseases usually progress slowly but can progress rapidly [27, 28]. In this cohort, those with degenerative disease comprised the second largest group, accounting for 20.8% of all RPD cases, and the prevalence from high to low was AD, DLB, neuronal intranuclear inclusion disease and FTLD. These data were in agreement with several studies, such as a German series that suggested that AD was the most common etiology of nonprion RPD, followed by FTLD and DLB [29]. However, another study pointed out that rpAD was better defined by a survival time less than 3 years rather than a rate of cognitive decline defined by MMSE scores [30]. The prevalence of rpAD varied among studies, with a prevalence of 10–30% reported among those with mild AD and 17.6% reported based on a large database [31]. However, remarkably, it was demanding to attach importance to vague complaints such as fatigue, sleep disturbance, depression, and behavioral changes that preceded the incidence of cognitive complaints.

The third largest group of RPD patients had toxic-metabolic diseases. Specifically, CO poisoning as a potentially treatable disorder was reported in a substantial number of patients in the present study and was the third most common disease after CJD and AD, characterized by an early onset of headache, general pain, consciousness impairment, seizure, general fatigue, limb shaking, and a late occurrence of cognitive decline, slow response, apathy, reduced active speech, reduced physical activity and so on [32, 33]. Considering its curability and reversibility, it was recommended to conduct an initial screening of CO poisoning. Similarly, various antibody-mediated autoimmune syndromes have gradually been recognized owing to the continuous development of antibody detection technology in recent years, and anti-AMPA2, anti-LG1, anti-GABAB receptor encephalitis and undetermined types of inflammation were demonstrated here. Inconsistent with previous studies indicating immune-mediated diseases as the cause in a majority of RPD patients [12, 34], this study observed a small population of inflammatory entities, which may have been due to an onset of psychiatric symptoms, consciousness impairment and recurrent seizure at that stage, rather than measurable cognitive impairment.

Another highlight of this study was exploring the prognostic factors of RPD. In this cohort, a compatible conclusion was eventually made: age and causation were the main prognostic factors. Specifically, neoplastic RPD participants were more likely to be in a longitudinal state of death or vegetative symptoms. Conversely, inflammatory and toxic/metabolic RPD participants were likely to have good outcomes. This phenomenon further strengthens the importance of a quick and accurate identification of RPD etiologies to specifically differentiate inflammatory or toxic/metabolic RPD from neoplastic or degenerative RPD.

There are some limitations in this study. This was a single-center study with a retrospective design that mainly included medical records and analyzed data from 2015 to 2019. Thus, some testing and clinical evaluation data were incomplete, and the relative sample size was insufficient to establish an appropriate prognostic model. However, it emerged from this study that RPD is a challenging category with diverse causative factors, and accurate and quick identification of RPD etiology, especially in treatable cases, will allow favorable outcomes, whereas a delayed diagnosis will result in poor outcomes. Thus, a practical approach is urgently needed, although misdiagnosis remains inevitable in a minority of patients. In this cohort, infectious, neurodegenerative and toxic-metabolic entities were the largest groups, while CJD, AD, CO poisoning, neurosyphilis and DLB were the most common diseases. Therefore, a recommended diagnostic framework of RPD etiology in line with local conditions was established here. Moreover, for outcome evaluations, RPD prognosis may depend on causation and age.

## Data Availability

The datasets generated during or analyzed during the current study available from the corresponding author on reasonable request.

## List of Abbreviations

ADC: Apparent diffusion coefficient
ADL: Activities of daily living scale
CJD: Creutzfeldt–Jakob disease
CO: Carbon monoxide
CRP: C-reactive protein
CSF: Cerebrospinal fluid
DLB: Dementia with Lewy bodies
DWI: Diffusion-weighted imaging
EEG: Electroencephalography
ESR: Erythrocyte sedimentation rate
FLAIR: Fluid-attenuated inversion recovery
FTLD: Frontotemporal lobar degeneration
GOS: Glasgow Outcome Scale
MMSE: Mini-Mental State Examination
MRI: Magnetic resonance imaging
PET: Positron emission tomography
rpAD: Rapidly progressive Alzheimer’s disease
RPD: Rapidly progressive dementia
SWI: Susceptibility weighted imaging
